# NUP98重排儿童急性髓系白血病患者行异基因造血干细胞移植的预后分析

**DOI:** 10.3760/cma.j.cn121090-20251109-00515

**Published:** 2026-05

**Authors:** 慧芳 王, 冠华 胡, 露 柏, 盼 锁, 枫 张, 英熹 左, 开彦 刘, 兰平 许, 昱 王, 于谦 孙, 新欣 贺, 晓辉 张, 晓军 黄, 翼飞 程

**Affiliations:** 北京大学人民医院，北京大学血液病研究所，国家血液系统疾病临床医学研究中心，造血干细胞移植北京市重点实验室，北京 100044 Peking University People's Hospital, Peking University Institute of Hematology, National Clinical Research Center for Hematologic Disease, Beijing Key Laboratory of Hematopoietic Stem Cell Transplantation, Beijing 100044, China

**Keywords:** 白血病，髓系，急性, 儿童, 异基因造血干细胞移植, NUP98重排, 预后, Leukemia, myeloid, acute, Pediatric, Allogeneic hematopoietic stem cell transplantation, NUP98 rearrangement, Prognosis

## Abstract

**目的:**

分析核孔蛋白98重排（NUP98-r）儿童急性髓系白血病（pAML）患者行异基因造血干细胞移植（allo-HSCT）的预后及allo-HSCT前后NUP98-r对预后的影响。

**方法:**

本研究为回顾性单臂观察性队列研究。纳入2018年4月至2024年10月在北京大学人民医院行allo-HSCT的NUP98-r pAML患者，收集其临床特征、allo-HSCT前后NUP98-r情况、复发和生存结局，并分析allo-HSCT前后NUP98-r对预后的影响。

**结果:**

31例行allo-HSCT的NUP98-r pAML患者中，男16例（51.6％），女15例（48.4％），中位年龄8（2～18）岁，中位随访时间35（6～66）个月。最常见的NUP98-r亚型为NUP98::NSD1（22例，71.0％），其次为NUP98::HOXA9（5例，16.1％）。18例（58.1％）合并FLT3-ITD突变，14例（45.2％）合并WT1突变。7例患者allo-HSCT后复发，复发中位时间6（3～48）个月。Ⅱ～Ⅳ级急性移植物抗宿主病100 d累积发生率为（35.4±8.6）％，慢性移植物抗宿主病3年累积发生率为（64.8±9.2）％。3年总生存（OS）率、无事件生存（EFS）率和累积复发率（CIR）分别为（74.0±9.6）％、（63.9±10.1）％、（21.3±8.7）％。allo-HSCT前NUP98-r阴性患者的3年EFS率高于NUP98-r阳性患者［（92.3±7.4）％对（42.3±15.3）％，*P*＝0.044］，NUP98-r阳性患者的3年CIR高于NUP98-r阴性患者［（34.8±14.6）％对0，*P*＝0.036］。allo-HSCT后前3个月NUP98-r阳性的患者后续均复发。

**结论:**

allo-HSCT是NUP98-r pAML有效的治疗方式，且allo-HSCT前NUP98-r阳性与更高的CIR和更低的EFS率相关，可通过治疗使NUP98-r转阴后行allo-HSCT。

## Abstract (en)

**Objective:**

To analyze the prognosis and impact of NUP98 rearrangements（NUP98-r）pre- and post-allogeneic hematopoietic stem cell transplantation（allo-HSCT）in patients with pediatric acute myeloid leukemia（pAML）with NUP98-r who underwent allo-HSCT.

**Methods:**

This was a retrospective, single-arm, observational cohort study. This study enrolled patients with pAML with NUP98-r who underwent allo-HSCT at Peking University People's Hospital from April 2018 to October 2024. The clinical characteristics, NUP98-r status pre- and post-allo-HSCT, relapse, and survival outcomes of the patients were collected. The impact of NUP98-r status pre- and post-allo-HSCT on prognosis was analyzed.

**Results:**

Among 31 patients with pAML with NUP98-r who underwent allo-HSCT, 16（51.6％）were males and 15（48.4％）were females, with a median age of 8 years（range: 2–18 years）. The median follow-up time was 35 months (range: 6–66 months). The most common NUP98-r subtype was NUP98::NSD1（22 patients，71.0％）, followed by NUP98::HOXA9（5 patients，16.1％）. Concurrent FLT3-ITD mutation was present in 18 patients（58.1％）and WT1 mutation in 14 patients（45.2％）. Seven patients relapsed post-allo-HSCT, with a median time to relapse of 6 months（range: 3–48 months）. The 100-day cumulative incidence of gradeⅡ–Ⅳ acute graft-versus-host disease（GVHD）was（35.4±8.6）％, and the 3-year cumulative incidence of chronic GVHD was（64.8±9.2）％. The 3-year overall survival, event-free survival（EFS）, and cumulative incidence of relapse（CIR）rates were（74.0±9.6）％,（63.9±10.1）％, and（21.3±8.7）％, respectively. The 3-year EFS was higher in patients who were negative for NUP98-r pre-allo-HSCT［（92.3±7.4）％］than in those positive for NUP98-r［（42.3±15.3）％］（*P*＝0.044）. The 3-year CIR was higher in patients positive for NUP98-r pre-allo-HSCT［（34.8±14.6）％］than in those negative for NUP98-r（0）（*P*＝0.036）. All patients who were positive for NUP98-r within the first 3 months post-allo-HSCT subsequently relapsed.

**Conclusion:**

Allo-HSCT is an effective method for treating NUP98-r pAML, and NUP98-r positivity pre-allo-HSCT is associated with higher CIR and lower EFS. Therefore, efforts should be made to achieve a negative NUP98-r status through treatment before undergoing allo-HSCT.

儿童急性髓系白血病（pAML）是一种罕见且异质性很强的血液肿瘤，占儿童白血病的15％～20％，其具有较高的耐药性和复发率[1]–[2]。尽管在过去30余年里，随着风险分层的优化、强化化疗、移植技术的改善和支持性护理的改进，pAML的长期生存率稳定在70％左右，仍有大约30％的患者会复发，且预后很差[3]。细胞遗传学和分子生物学特征是影响pAML预后分层的重要因素。

核孔蛋白98（NUP98）位于染色体11p15，其N端结构域和伴侣基因C端区域重排（NUP98-r）形成融合蛋白，构成核孔蛋白复合物[4]，在介导RNA分子在细胞质和细胞核之间的选择性转运中发挥重要作用，同时也参与转录调控和有丝分裂[5]–[7]。自1996年NUP98::HOXA9被发现以来，越来越多的NUP98伴侣基因被认识，目前已经在髓系肿瘤中发现了30多种NUP98的伴侣基因[8]–[9]。NUP98-r在pAML中的发生率为5％～10％，是pAML常见的融合基因类型[7]–[8],[10]–[13]。NUP98-r AML患者复发率高，完全缓解率（CR）低，总生存（OS）和无事件生存（EFS）率低[8],[12],[14]–[17]，异基因造血干细胞移植（allo-HSCT）可能是改善其预后的治疗方式[17]。目前探讨NUP98-r pAML行allo-HSCT预后的研究较少。本研究旨在分析NUP98-r pAML行allo-HSCT的预后及allo-HSCT前后NUP98-r对预后的影响。

## 病例与方法

1. 研究对象：本研究为回顾性单臂观察性队列研究。纳入2018年4月至2024年10月在北京大学血液病研究所行allo-HSCT的NUP98-r pAML（≤18岁）患者，为连续病例，使用2016年WHO髓系肿瘤和急性白血病分类标准[18]对患者进行诊断。本研究得到北京大学人民医院伦理委员会批准（批件号：2025PHD013-001）。

2. NUP98-r融合基因检测：所有患者均使用骨髓标本，采用基于TaqMan的RQ-PCR方法检测NUP98-r融合基因，内参基因为ABL。NUP98-r融合基因表达水平（％）＝NUP98-r融合基因拷贝数/ABL基因拷贝数×100％。NUP98-r转录水平>0定义为阳性，其敏感性为10^−5^。

3. 移植方案：本研究中纳入的患者接受清髓预处理。单倍体异基因造血干细胞移植（haplo-HSCT）：地西他滨200 mg·m^−2^·d^−1^，静脉滴注，−11、−10 d（仅allo-HSCT前NUP98-r阳性患者使用）；阿糖胞苷（Ara-C）4 g·m^−2^·d^−1^，静脉滴注，−9 d；白消安（BU）3.2 mg·kg^−1^·d^−1^，静脉滴注，−8～−6 d；环磷酰胺（CTX）1.8 g·m^−2^·d^−1^，−5、−4 d；司莫司汀（Me-CCNU）250 mg/m^2^，−3 d；兔抗人胸腺细胞免疫球蛋白（rATG）2.5 mg·kg^−1^·d^−1^，−5～−2 d。同胞全相合HSCT（MSD-HSCT）：地西他滨、阿糖胞苷、白消安、CTX、司莫司汀用法同上，不用rATG。所有患者均接受经过G-CSF（5 µg·kg^−1^·d^−1^，连续5 d）刺激的外周血干细胞和（或）骨髓输注。所有患者均使用环孢素A+霉酚酸酯+短程甲氨蝶呤预防移植物抗宿主病（GVHD）。

4. 定义：CR定义为骨髓中原始细胞<5％，三系造血恢复并且没有白血病细胞髓外浸润；OS时间定义为移植至死亡或随访结束的时间；EFS时间定义为移植至首次事件发生的时间，包括复发、任何原因死亡或第二种恶性肿瘤。

5. 随访：本研究通过查阅门诊病历、住院病历及电话进行随访，随访截止时间为2025年5月9日，没有患者失访。

6. 统计学处理：使用SPSS26.0进行统计学分析。患者临床特征采用描述性统计，分类变量采用比例描述，连续性变量采用中位数（范围）描述。使用Kaplan-Meier法和对数秩检验进行统计学分析，*P*<0.05为差异有统计学意义。使用GraphPad Prism 10.6.0制作生存曲线和泳道图。

## 结果

1. 患者特征：本研究纳入31例NUP98-r pAML患者，其中男16例（51.6％），女15例（48.4％），中位年龄8（2～18）岁。截至末次随访，中位随访时间35（6～66）个月。如表1所示，最常见的FAB分型为M_5_，其次为M_2_和M_4_。最常见的NUP98-r亚型是NUP98::NSD1，共22例，占71.0％，其他依次为NUP98::HOXA9 5例（16.1％），NUP98::PRRX2、NUP98::HMGB3、NUP98::TNRC18、NUP98::H1-8各1例（3.2％）。18例（58.1％）合并FLT3-ITD突变，14例（45.2％）合并WT1突变，10例（32.3％）合并NRAS突变，4例（12.9％）合并RUNX1突变，3例（9.7％）合并KRAS突变，3例（9.7％）合并EVI1突变。在22例NUP98::NSD1患者中，有17例（77.3％）患者发生FLT3-ITD突变。5例（16.1％）患者allo-HSCT前流式细胞术可检测残留病（MRD）阳性。29例患者allo-HSCT前有NUP98-r结果，16例（55.2％）阳性，13例（44.8％）阴性。28例（90.3％）患者在第1次完全缓解（CR_1_）期行allo-HSCT，2例（6.5％）患者在CR_2_期行allo-HSCT，1例（3.2％）患者在CR_3_期行allo-HSCT。

**表1:** 31例行异基因造血干细胞移植的NUP98重排儿童AML患者临床特点

特征	数值
性别［例（％）］	
男	16（51.6）
女	15（48.4）
年龄［岁，*M*（范围）］	8（2～18）
FAB分类［例（％）］	
M_2_	11（35.5）
M_4_	5（16.1）
M_5_	12（38.7）
MDS转化AML［例（％）］	1（3.2）
治疗相关AML［例（％）］	1（3.2）
初诊白细胞［×10^9^/L，*M*（范围）］	36（2～496）
NUP98融合基因类型［例（％）］	
NUP98::NSD1	22（71.0）
NUP98::HOXA9	5（16.1）
NUP98::PRRX2	1（3.2）
NUP98::HMGB3	1（3.2）
NUP98::TNRC18	1（3.2）
NUP98::H1-8	1（3.2）
基因突变［例（％）］	
FLT3-ITD	18（58.1）
WT1	14（45.2）
NRAS	10（32.3）
RUNX1	4（12.9）
KRAS	3（9.7）
EVI1	3（9.7）
CEBPA单突变	1（3.2）
FLT3-TKD	1（3.2）
GATA2	1（3.2）
BCOR	1（3.2）
移植前流式细胞术MRD［例（％）］	
阳性	5（16.1）
阴性	26（83.9）
移植前NUP98重排［例（％）］^a^	
阳性	16（55.2）
阴性	13（44.8）
移植前状态［例（％）］	
CR_1_	28（90.3）
CR_2_	2（6.5）
CR_3_	1（3.2）
移植类型［例（％）］	
同胞全相合	3（9.7）
单倍体	28（90.3）
供者和受者关系［例（％）］	
父	24（77.4）
母	2（6.5）
兄	4（12.9）
姐	1（3.2）
干细胞来源［例（％）］	
PB+BM	5（16.1）
PB	26（83.9）
单个核细胞计数［×10^8^/kg，*M*（范围）］	10.6（5.8～15.5）
CD34^+^细胞计数［×10^6^/kg，*M*（范围）］	5.5（1.7～20.5）
FLT3抑制剂［例（％）］^b^	
吉瑞替尼	9（64.3）
索拉非尼	5（35.7）

**注** AML：急性髓系白血病；MDS：骨髓增生异常综合征；MRD：可检测残留病；CR_1～3_：第1～3次完全缓解；PB：外周血；BM：骨髓；^a^29例患者有移植前NUP98重排结果；^b^14例患者移植后使用FLT3抑制剂

2. 植入和GVHD：MSD-HSCT 3例（9.7％），haplo-HSCT 28例（90.3％）。1例患者在回输造血干细胞前死亡，余30例患者白细胞和血小板均植入，白细胞中位植入时间12（9～22）d，血小板中位植入时间11（8～30）d。30例患者Ⅱ～Ⅳ级急性GVHD（aGVHD）100 d累积发生率（35.4±8.6）％，Ⅲ～Ⅳ级aGVHD 100 d累积发生率（9.7±5.3）％。移植后100 d仍存活29例患者中，慢性GVHD（cGVHD）的3年累积发生率为（64.8±9.2）％，广泛型cGVHD的3年累积发生率为（35.0±9.8）％。

3. 复发和非复发死亡：截至末次随访，7例患者allo-HSCT后复发，中位复发时间6（3～48）个月。6例患者血液学复发，另外1例患者中枢神经系统复发，随后血液学复发。本研究中7例患者死亡，3例死于复发，4例为非复发死亡。在非复发死亡的患者中，2例死于cGVHD，1例死于弥漫性肺泡出血，1例（神经母细胞瘤治疗后AML）死于预处理期间脓毒血症。

4. OS、EFS和累计复发率（CIR）：分析31例患者的生存情况，3年OS率、EFS率和CIR分别为（74.0±9.6）％、（63.9±10.1）％、（21.3±8.7）％（图1A～C）。不同NUP98伙伴基因对pAML患者移植后的OS、EFS无明显影响（图1D、E），NUP98::HOXA9患者的3年CIR［（60.0±21.9）％］高于NUP98::NSD1［（82.7±9.4）％］和NUP98-Others患者（0）（*P*＝0.039）（图1F）。29例移植前有NUP98-r检测结果的患者中，NUP98-r阴性和阳性患者的3年OS率分别为（92.3±7.4）％和（58.0±14.8）％（*P*＝0.172），差异无统计学意义（图2A）。allo-HSCT前NUP98-r阴性患者的3年EFS率为（92.3±7.4）％，高于NUP98-r阳性患者的（42.3±15.3）％（*P*＝0.044）（图2B）。移植前NUP98-r阳性患者的3年CIR为（34.8±14.6）％，高于NUP98-r阴性患者（0）（*P*＝0.036）（图2C）。是否合并FLT3-ITD突变对NUP98::NSD1 pAML患者的OS、EFS和CIR无明显影响（**扫描本文首页二维码查阅附图1**）。

**图1:**
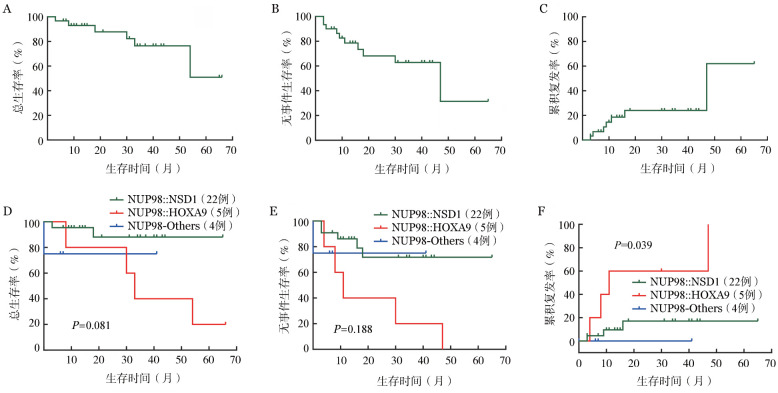
31例行异基因造血干细胞移植的NUP98重排（NUP98-r）儿童急性髓系白血病（pAML）患者的预后 **A** 总生存曲线；**B** 无事件生存曲线；**C** 累积复发曲线；**D～F** 不同NUP98伙伴基因对pAML患者的总生存、无事件生存和累积复发的影响

**图2:**
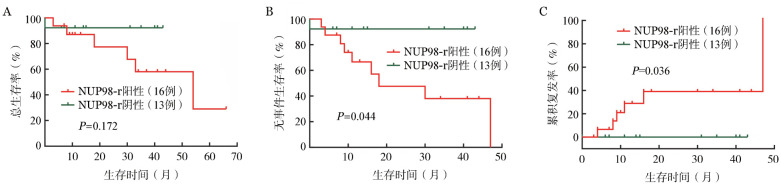
异基因造血干细胞移植前NUP98重排（NUP98-r）是否阳性对儿童急性髓系白血病患者患者总生存（A）、无事件生存（B）和累积复发（C）的影响

5. FLT3抑制剂对合并FLT3-ITD突变NUP98-r pAML患者预后的影响：18例NUP98-r患者合并FLT3-ITD突变，共14例患者allo-HSCT后使用FLT3抑制剂，其中9例使用索拉菲尼，5例使用吉瑞替尼。12例患者在allo-HSCT后1～2个月开始使用FLT3抑制剂，2例患者在allo-HSCT后NUP98-r阳性后开始使用FLT3抑制剂，用至移植后2年或者出现复发。是否使用FLT3抑制剂对合并FLT3-ITD突变的NUP98-r pAML患者OS、EFS和CIR无明显影响（**扫描本文首页二维码查阅附图2**）。

6. 移植后早期NUP98-r对预后的影响：30例患者在allo-HSCT后第1、2和3个月检测了NUP98-r水平。allo-HSCT后第1个月，1例患者NUP98-r阳性；第2个月，3例患者NUP98-r阳性；第3个月，4例患者NUP98-r阳性。allo-HSCT后3个月内NUP98-r阳性的患者，后续均血液学复发（图3）。

**图3:**
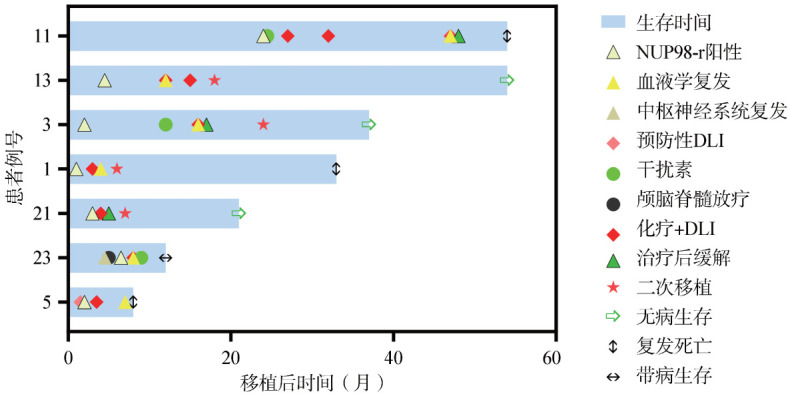
异基因造血干细胞移植（allo-HSCT）后复发患者的治疗和结局 **注** 例1 allo-HSCT后1、2和3个月NUP98重排（NUP98-r）均阳性，例3和例5 allo-HSCT后2个月和3个月NUP98-r均阳性，例21 allo-HSCT后3个月NUP98-r阳性；DLI：供者淋巴细胞输注

7. 复发后的治疗和转归：6例allo-HSCT后单纯血液学复发患者行化疗+供者淋巴细胞输注（DLI），3例CR，2例CR后行二次allo-HSCT未再复发并存活，另1例未行二次allo-HSCT的患者在CR后再次出现复发；3例行化疗+DLI后未缓解，1例死亡，2例行二次allo-HSCT后CR，其中1例再次出现复发死亡，1例发生广泛型GVHD，持续缓解（图3）。1例患者中枢神经系统复发后行全颅脑脊髓放疗后CR，随后出现了血液学复发，化疗+DLI后缓解，后反复分子学复发，予干扰素治疗，发生重度aGVHD，截至随访日期仍带病存活（图3）。另3例患者移植后出现NUP98-r阳性，2例患者干扰素治疗后转阴，1例化疗+DLI后转阴，至随访日期NUP98-r仍持续阴性。

## 讨论

本研究纳入31例行allo-HSCT的NUP98-r pAML患者，以M_2_和M_4_/M_5_亚型为主。NUP98-r在pAML中最常见的亚型是NUP98::NSD1，占71.0％，与既往报道相似[8]。本研究以haplo-HSCT为主，占90.3％，Ⅱ～Ⅳ级aGVHD和Ⅲ～Ⅳ级aGVHD发生率与本单位既往研究相符，而cGVHD和广泛型cGVHD发生率高于既往研究[19]，可能是allo-HSCT后DLI的患者较多。Yan等[20]的研究证实DLI会增加cGVHD风险，降低CIR，提高生存。

Bertrums等[8]报道了目前病例数最多的NUP98-r pAML队列，涉及160例NUP98-r pAML患者，其中104例NUP98::NSD1，32例NUP98::KDM5A，NUP98-r患者的5年OS率明显比对照组差（35％对64％，*P*<0.001），NUP98::NSD1亚组OS和EFS更差，其CIR明显升高。另一项57例NUP98-r pAML队列中，有11例患者行allo-HSCT，5年OS率和EFS率分别为48.1％和32.4％，比非NUP98-r组差[16]，同样的结论在其他研究中也得到了证实[11]–[12],[21]–[22]。Yuan等[23]报道了56例NUP98-r的成人AML，其2年OS率、DFS率和CIR分别为73％、69.8％和28.4％。本研究3年OS率和EFS率分别为74％和63.9％，3年CIR为21.3％，与Yuan等的报道相似。我们认为allo-HSCT改善了NUP98-r患者的预后，既往也有研究证实移植是改善NUP98-r AML患者生存的方式[17]。NUP98::HOXA9与AML更差的无复发生存有关[24]–[26]，我们也发现NUP98::HOXA9患者有更高的CIR，提示NUP98::HOXA9阳性的AML患者需要更积极和强力的治疗。

本研究显示FLT3-ITD对NUP98::NSD1 pAML患者预后没有明显影响，这与既往NUP98::NSD1合并FLT3-ITD突变时预后更差[16],[27]的报道不符，可能是allo-HSCT及allo-HSCT后FLT3-ITD抑制剂维持治疗（14/18）克服了这一不利因素，也可能因病例数少而没有显现出差异。多项研究和Meta分析已证实，allo-HSCT后FLT3-ITD抑制剂（索拉菲尼或吉瑞替尼）维持治疗改善FLT3突变AML患者的生存并减少复发，且不增加非复发死亡率、aGVHD和cGVHD的发生率[28]–[31]。本研究allo-HSCT后FLT3-ITD抑制剂维持治疗对OS、EFS和CIR没有明显影响，可能与病例数少有关。

既往研究和Meta分析均显示allo-HSCT前MRD阳性对预后有不利影响[19],[32]。本研究结果显示，allo-HSCT前NUP98-r阳性患者EFS率更低，CIR更高，提示我们allo-HSCT前通过积极治疗使NUP98-r转阴可能是改善NUP98-r患者预后的可行办法。本研究中allo-HSCT后3个月内NUP98-r阳性的患者后续均复发，尚亚可等[33]的研究显示NUP98::NDS1融合基因可用于评估该类AML的MRD状态，并且移植后NUP98::NSD1阳性患者复发率高，预后差。Yuan等[23]研究显示allo-HSCT后1个月NUP98-r阳性预测了更高的CIR，更低的OS和DFS。加强allo-HSCT后早期NUP98-r的监测，如果allo-HSCT后3个月内出现NUP98-r阳性，应给予积极的干预，可尝试化疗+DLI或者menin抑制剂等[23],[34]。

本研究中7例allo-HSCT后复发的患者均行化疗+DLI治疗，半数患者再次获得CR，其中2例进行二次allo-HSCT的患者持续缓解，余2例未桥接二次移植的患者，1例复发死亡，1例合并广泛型cGVHD带病生存。化疗+DLI虽然可以使部分复发患者再次获得CR，但并不持久，桥接二次移植可能是获得长期生存的有效治疗方式。

本研究为单中心回顾性研究，样本量少，缺乏单纯化疗的患者作为对照。日后需要设计前瞻性、多中心和大样本量的研究来阐明NUP98-r pAML患者allo-HSCT的预后及其影响因素。

总之，allo-HSCT是NUP98-r pAML有效的治疗方式，且allo-HSCT前NUP98-r阳性与更高的CIR和更低的EFS率相关，可通过治疗使NUP98-r转阴后行allo-HSCT。

## Supplementary Material


