# Supplementary figures and images for: Prognostic analysis of NUP98 rearrangement in patients with pediatric acute myeloid leukemia undergoing allogeneic hematopoietic stem cell transplantation
Source: Zhonghua Xue Ye Xue Za Zhi. 2026 May;47(5):458–64. [Article in Chinese] doi: 10.3760/cma.j.cn121090-20251109-00515 (PMC13416544; doi:10.3760/cma.j.cn121090-20251109-00515)

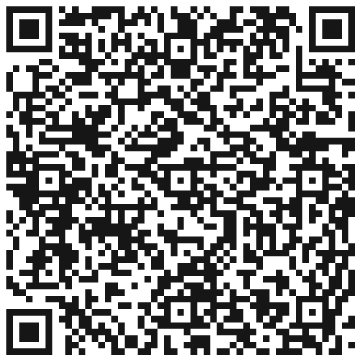

Supplement: Supplementary file 1 [file cjh-47-05-458-g004.tif]
